# Association Between Perioperative Daprodustat Use and Early Posttransplant Graft Function in Living Donor Kidney Transplantation: A Propensity Score-Matched Retrospective Study

**DOI:** 10.1016/j.curtheres.2026.100837

**Published:** 2026-06-20

**Authors:** Tomohiro Shigematsu, Soichiro Tajima, Hiroshi Noguchi, Takashi Ogino, Keizo Kaku, Takeshi Hirota, Mayako Uchida

**Affiliations:** a Department of Pharmacy, Kyushu University Hospital, Fukuoka, Japan; b Department of Surgery and Oncology, Graduate School of Medical Sciences, Kyushu University, Fukuoka, Japan; c Department of Clinical Pharmacology and Biopharmaceutics, Graduate School of Pharmaceutical Sciences, Kyushu University, Fukuoka, Japan

**Keywords:** Graft function, Hypoxia-inducible factor (HIF) prolyl hydroxylase (PHD) inhibitor, Inflammation, Ischemia-reperfusion injury, Kidney transplantation

## Abstract

**Background:**

Hypoxia-inducible factor prolyl hydroxylase (HIF-PH) inhibitors, such as daprodustat, have demonstrated nephroprotective effects in preclinical models. However, their potential benefits in living donor kidney transplantation (LDKT) remain unclear. This study aimed to evaluate the effects of daprodustat on early posttransplant graft functions in LDKT.

**Methods:**

This single-center study evaluated LDKT recipients transplanted between August 2019 and November 2023. Patients were divided into the daprodustat group, who received the drug from preoperative Day 1 to postoperative Day 7, and the control group, who did not receive HIF-PH inhibitors during this period. Propensity score matching yielded 32 patients per group.

**Results:**

The daprodustat group exhibited better graft function, reflected by higher estimated glomerular filtration rates in the early posttransplant period (mean ± standard deviation: Week 1, 53.3 ± 16.1 vs control 41.9 ± 17.9 mL/min/1.73 m²; *P* = 0.001; Week 2, 55.3 ± 17.6 vs 46.0 ± 15.1; *P* = 0.008). The incidence of slow graft function was significantly lower in the daprodustat group (0.0% vs 12.5%, *P* = 0.039). No delayed graft function or thrombotic events occurred, and rejection did not differ between groups.

**Conclusions:**

These findings raise the possibility that perioperative daprodustat use may be associated with improved early graft function in LDKT, warranting further investigation.

## Introduction

Kidney transplantation (KT) is the optimal treatment for patients with end-stage kidney disease (ESKD), improving quality of life and survival over dialysis. However, donor organ shortage limits transplantation opportunities.^1^ To address this issue, a key strategy is to improve graft function and survival, thereby reducing the need for retransplantation. Another strategy is the use of marginal kidneys to expand the donor pool, though this increases the risk of poor early graft function.^2^

During the peri‑transplant period, the kidney grafts face multiple insults, including ischemia-reperfusion injury (IRI), immune system disorders, immunosuppressant nephrotoxicity, and surgical stress.^2^^,^^3^ Among these, IRI is inevitable and cause tubular cell death, inflammation, vascular injury, and immune activation.^4^ These responses impair early graft function and contribute to rejection, leading to long-term graft dysfunction and loss.^2^, ^3^, ^4^ Poor early graft function, including delayed graft function (DGF) and its milder form, slow graft function (SGF), negatively impacts long-term graft outcomes.^5^, ^6^, ^7^ The incidence of poor early graft function has been reported at 10% to 20% in living donor KT (LDKT).^6^^,^^7^ Moreover, DGF occurred in 26.3% of adult KT recipients in the United States in 2022, and its incidence has increased over the past decade.^1^ Therefore, IRI is a key therapeutic target, and multiple strategies have been investigated.^2^ The combination of these approaches is expected to improve graft function and survival.

Hypoxia-inducible factor prolyl hydroxylase (HIF-PH) inhibitors are novel oral agents for anemia in patients with chronic kidney disease (CKD), demonstrating efficacy in both dialysis and nondialysis patients.^8^ These agents stabilize the HIF complex and stimulate endogenous erythropoietin production, even in patients with ESKD.^8^ Although KT recipients have been excluded from clinical trials,^8^ HIF-PH inhibitors are now used for the treatment of posttransplant anemia, with emerging reports on their efficacy and safety.^9^^,^^10^

HIF regulates multiple target genes involved in cellular adaptation to hypoxia and exert nephroprotective effects.^11^ Experimental studies, including KT models, have suggested that activation of the HIF pathway attenuates IRI and improves graft outcomes.^11^, ^12^, ^13^ Furthermore, observational studies in human KT have reported an association between increased HIF-1α expression and better graft function.^14^^,^^15^ However, clinical evidence regarding the potential early posttransplant nephroprotective effects of HIF-PH inhibitors in human KT remains uncertain and speculative.^16^

Therefore, we hypothesized that HIF-PH inhibitors would prevent early graft dysfunction in LDKT. To test this hypothesis, we conducted a retrospective investigation and analysis of patients who received daprodustat during the perioperative period of LDKT.

## Materials and Methods

### Study design

This was a single-center, retrospective, observational cohort study conducted at Kyushu University Hospital, Fukuoka, Japan. The study was conducted in accordance with the Declaration of Helsinki and was approved by the Kyushu University Institutional Review Board for Clinical Research (IRB No. 24234). Because of the retrospective nature of the study, the requirement for written informed consent was waived. Instead, an opt-out consent process was implemented, whereby patients were provided with the opportunity to refuse participation through publicly available information.

### Study population

This study screened 220 recipients who underwent LDKT between August 2019 and November 2023 and received immunosuppressive therapy with tacrolimus (TAC), mycophenolate mofetil (MMF), methylprednisolone (mPSL), and basiliximab. The exclusion criteria were as follows: recipients with preformed donor-specific anti-human leukocyte antigen (HLA) antibodies, individuals undergoing retransplantation, recipients followed up for less than 1 year after transplantation, and recipients under 18 years of age. Eligible patients were divided into a daprodustat group and a control group. The daprodustat group consisted of patients who received daprodustat daily from preoperative Day 1 to postoperative Day 7. At our institution, following its introduction in Japan in August 2021, daprodustat was prescribed at the discretion of the attending physician for patients undergoing LDKT, based primarily on hemoglobin levels at admission. Treatment was generally initiated at hemoglobin levels <11 g/dL in patients with nondialysis-dependent CKD or peritoneal dialysis and <10 g/dL in those receiving hemodialysis, according to the prescribing information. Dosing was based on the prescribing information and determined by physicians, ranging from 2 to 8 mg/day. Patients with a history of thrombosis did not receive daprodustat. The control group included all remaining patients, unless they had received HIF-PH inhibitors during this period. Propensity score matching (PSM) was performed to adjust baseline characteristics between the groups.

### Data collection

The baseline characteristics were collected at the time of transplantation. Clinical data, including recipient and donor characteristics, transplantation details, laboratory data, and kidney graft biopsy results, were retrospectively collected from electronic medical records. The estimated glomerular filtration rate (eGFR) was calculated using the appropriate equation for Japanese CKD patients.^17^ DGF was defined as the need for dialysis within the first week after transplantation, and SGF was defined as a serum creatinine level greater than 3.0 mg/dL on postoperative Day 5 without dialysis.^6^^,^^7^^,^^18^ In accordance with hospital protocol, kidney biopsies were performed at 3 months and 1 year postoperatively. The neutrophil-to-lymphocyte ratio (NLR), a marker reflecting the state of inflammation, was calculated by dividing the absolute neutrophil count by the absolute lymphocyte count.^19^

### Immunosuppressive protocol

TAC, MMF, and mPSL were administered orally as immunosuppressive agents. TAC dosing was adjusted by therapeutic drug monitoring to maintain a uniform target trough concentration range in all patients. For ABO-compatible recipients, oral immunosuppressive agents were initiated preoperative Day 7. ABO-incompatible recipients began oral immunosuppressive agents on preoperative Day 14, followed by rituximab and plasma exchange before transplantation. All patients who underwent LDKT were administered basiliximab (20 mg) at the time of surgery and on postoperative Day 4.

### Statistical analysis

PSM was performed using nearest-neighbor matching in a 1:1 ratio with a calliper width of 0.2. The matching variables included recipient sex, recipient body mass index, preemptive transplantation status, duration of dialysis, number of HLA mismatches, ABO incompatibility, preoperative recipient hemoglobin levels, donor age, and total ischemia time. Results are expressed as mean ± standard deviation for normally distributed variables or as median (interquartile range) otherwise. Categorical variables are expressed as frequencies (percentages). Mean bivariate differences between the two groups were assessed using the Student’s *t* test for normally distributed continuous variables and the Wilcoxon rank-sum test for ordinal variables. For continuous variables that were not normally distributed, median differences were compared using the Wilcoxon rank-sum test. Categorical variables were compared using the *χ*² test or Fisher’s exact test. Longitudinal changes in repeated measurements were analyzed using linear mixed-effects models (LMM), including group, time, and their interaction as fixed effects. Post hoc comparisons were performed using Tukey’s honestly significant difference test to identify differences between groups at individual time points. The area under the curve (AUC) of the NLR was calculated using the trapezoidal rule. All statistical analyses were performed using JMP software (version 17; SAS Institute Inc., Cary, North Carolina). All tests were two-sided, and a *P* value of < 0.05 was considered statistically significant.

## Results

### Baseline characteristics

**Figure 1:**
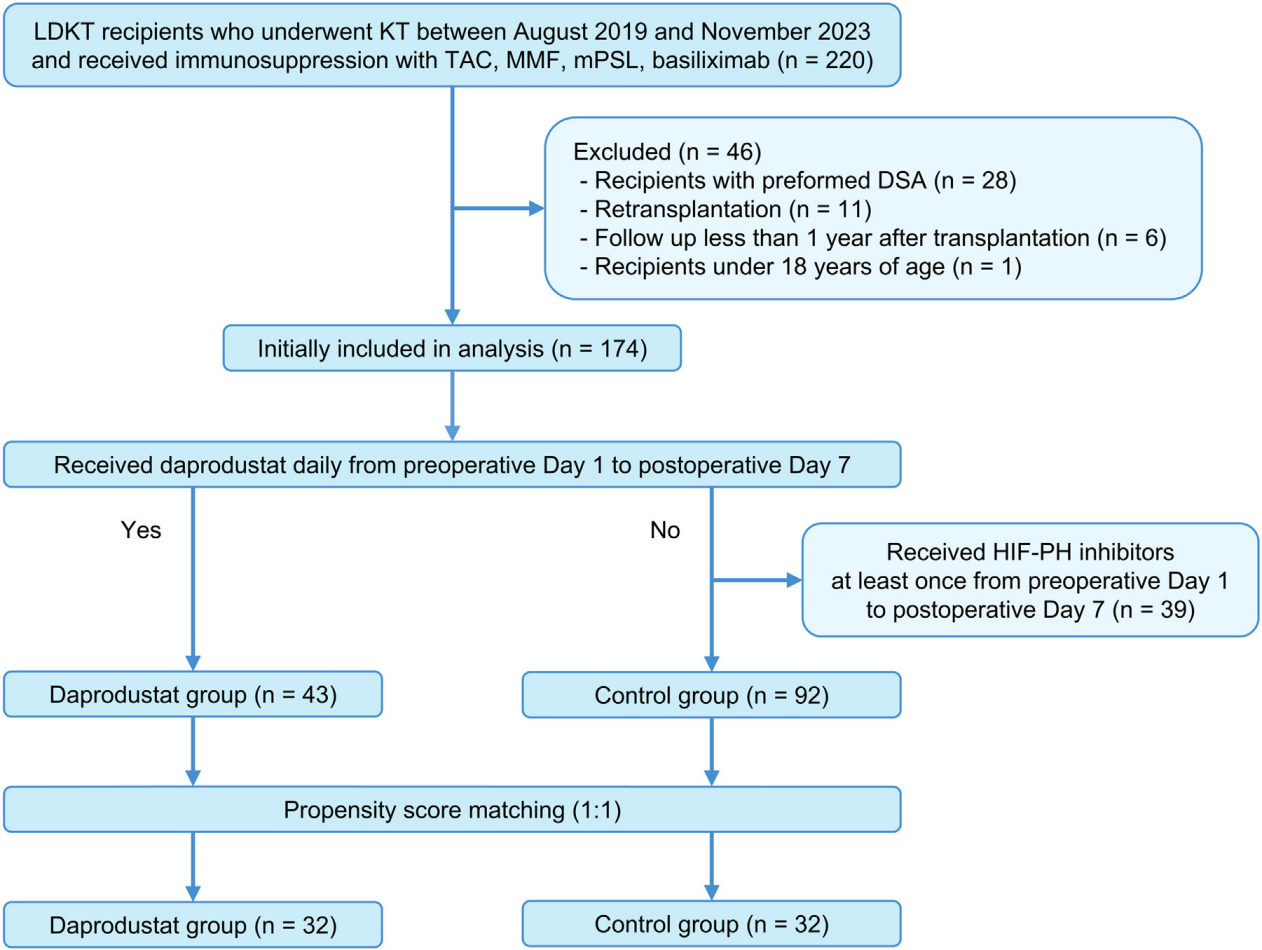
Flow chart of the study participants. DSA, donor-specific anti-HLA antibody; HIF-PH, hypoxia-inducible factor prolyl hydroxylase; KT, kidney transplantation; LDKT, living donor kidney transplantation; MMF, mycophenolate mofetil; mPSL, methylprednisolone; TAC, tacrolimus.

Of the 220 recipients screened, 174 met the eligibility criteria. Among them, 135 recipients were included in the final analysis, and 32 matched pairs were obtained after PSM (Figure 1). Table shows the baseline characteristics before and after PSM. Prior to PSM, significant differences between groups were observed in preemptive transplantation status (*P* = 0.005), duration of dialysis (*P* = 0.001), preoperative recipient hemoglobin level (*P* = 0.027), use of erythropoiesis-stimulating agents before transplantation (*P* = 0.049), cold ischemia time (*P* = 0.004), and total ischemia time (*P* = 0.005). After PSM, no significant differences were observed between the two groups. Preoperative kidney function was also comparable, and even among recipients of preemptive KT, it was essentially identical (*n* = 19 per group; daprodustat 8.8 ± 2.3 vs control 8.7 ± 1.8 mL/min/1.73 m^2^; *P* = 0.783).

**Table:** Baseline characteristics before and after propensity score matching.

Variables	Before matching (*n* = 135)			After matching (*n* = 64)		
	Daprodustat group (*n* = 43)	Control group (*n* = 92)	*P* value	Daprodustat group (*n* = 32)	Control group (*n* = 32)	*P* value
**Recipient factors**						
Age, mean ± SD, year	50.7 ± 11.4	50.7 ± 13.4	0.999	50.0 ± 12.1	51.2 ± 12.8	0.697
Sex, male: female	27: 16	69: 23	0.145	22: 10	26: 6	0.248
BMI, mean ± SD, kg/m^2^	22.6 ± 3.5	23.7 ± 3.8	0.082	23.0 ± 3.4	23.3 ± 3.6	0.783
Preoperative eGFR, mean ± SD, mL/min/1.73 m^2^	6.9 ± 2.8	6.2 ± 2.5	0.136	7.2 ± 2.8	6.9 ± 2.7	0.591
Pre-emptive transplantation, *n* (%)	28 (65.1)	36 (39.1)	0.005	19 (59.4)	19 (59.4)	1.000
Duration of dialysis, median (IQR), month	0 (0–4)	6 (0–31)	0.001	0 (0–5.8)	0 (0–12.5)	0.717
HLA mismatches, mean ± SD, *n*	3.1 ± 1.5	3.1 ± 1.6	0.983	2.9 ± 1.5	3.4 ± 1.5	0.217
ABO incompatibility, *n* (%)	10 (23.3)	35 (38.0)	0.090	10 (31.3)	8 (25.0)	0.578
Rituximab, *n* (%)	11 (25.6)	39 (42.4)	0.060	11 (34.4)	9 (28.1)	0.590
Plasmapheresis, median (IQR), times	0 (0–0)	0 (0–2)	0.082	0 (0–2)	0 (0–1)	0.720
Preoperative Hb level, mean ± SD, g/dL	10.6 ± 1.5	11.2 ± 1.3	0.027	10.6 ± 1.6	10.7 ± 1.5	0.793
Use of ESAs before transplantation, *n* (%)	24 (55.8)	67 (72.8)	0.049	17 (53.1)	19 (59.4)	0.614
Primary disease			0.869			0.534
Glomerular disease	10	18		6	4	
Polycystic disease	3	7		2	5	
Diabetes mellitus	10	31		9	8	
Hypertension/nephrosclerosis	4	7		3	1	
IgA nephropathy	8	11		6	4	
Other	6	14		4	9	
Unknown	2	4		2	1	
**Donor factors**						
Age, mean ± SD, year	55.6 ± 12.0	54.3 ± 12.2	0.574	57.0 ± 11.7	55.8 ± 12.4	0.680
Sex, male: female	15: 28	27: 65	0.517	8: 24	7: 25	0.768
BMI, mean ± SD, kg/m^2^	23.4 ± 2.9	22.7 ± 3.3	0.250	23.0 ± 2.8	22.8 ± 2.7	0.770
Preoperative eGFR, mean ± SD, mL/min/1.73 m^2^	78.5 ± 14.4	81.0 ± 18.0	0.420	78.6 ± 14.6	78.4 ± 16.4	0.950
**Operative factors**						
Warm ischaemia time, median (IQR), min	5.0 (4.0–6.0)	5.0 (4.0–6.0)	0.793	4.0 (4.0–6.0)	4.5 (3.0–5.8)	0.278
Cold ischaemia time, median (IQR), min	183 (153–258)	144 (116–214)	0.004	183 (140–238)	179 (123–242)	0.747
Total ischaemia time, median (IQR), min	189 (160–266)	151 (121–221)	0.005	188 (146–242)	184 (126–245)	0.763
Re warming time, median (IQR), min	62 (52–69)	57 (42–69)	0.103	60 (51–71)	58 (47–71)	0.587
Blood loss of recipients, median (IQR), mL	150 (111–281)	146 (80–236)	0.277	149 (90–262)	146 (80–258)	0.962

BMI, body mass index; eGFR, estimated glomerular filtration rate; ESAs, erythropoiesis stimulating agents; Hb, hemoglobin; HLA, human leukocyte antigen; IQR, interquartile range; SD, standard deviation.

### Graft function after transplantation

**Figure 2:**
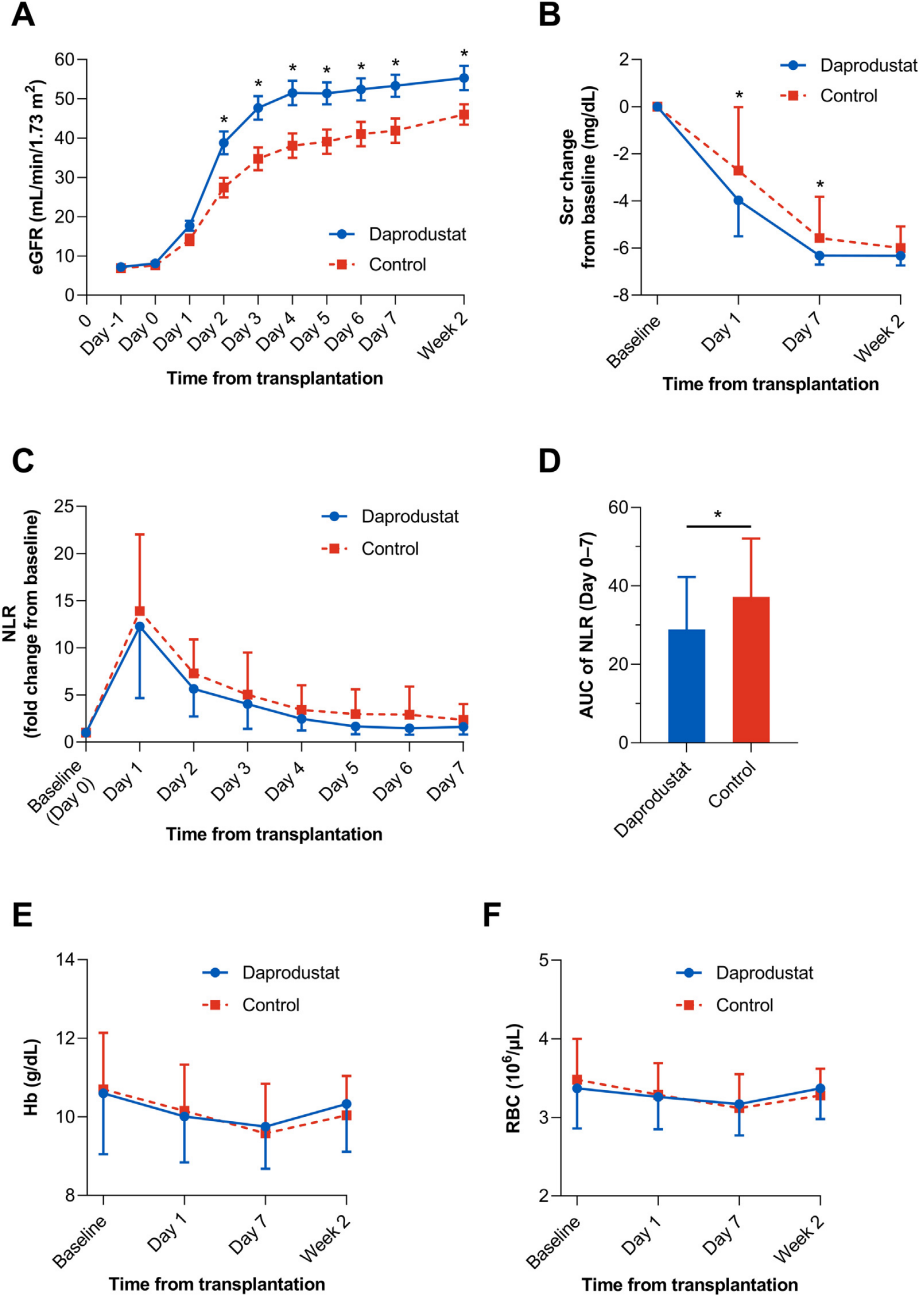
Changes in graft function, inflammatory marker, and hematological parameters after kidney transplantation. (A) estimated glomerular filtration rate (eGFR). (B) serum creatinine (Scr). (C) neutrophil-to-lymphocyte ratio (NLR). (D) area under the curve (AUC) of NLR. (E) hemoglobin (Hb). (F) red blood cell (RBC). eGFR values are presented as mean ± SEM, and all other values as mean ± SD. Significant group-by-time interactions were observed for eGFR (*P* < 0.001) and Scr change (*P* = 0.031) using a linear mixed-effects model with Tukey’s post hoc test. NLR values are expressed as fold change from baseline (Day 0), and the AUC of NLR was calculated from baseline to Day 7. No significant group-by-time interactions were observed for Hb (*P* = 0.916) or RBC (*P* = 0.796). *P < 0.05. AUC, area under the curve; eGFR, estimated glomerular filtration rate; Hb, hemoglobin; NLR, neutrophil-to-lymphocyte ratio; RBC, red blood cells; Scr, serum creatinine; SD, standard deviation; SEM, standard error of the mean.

The LMM showed a significant group-by-time interaction for eGFR (*P* < 0.001), indicating that the longitudinal eGFR trajectories differed between groups (Figure 2A). Post hoc analyses showed that eGFR was significantly higher in the daprodustat group during the early posttransplant period, particularly from postoperative Day 2 to Week 2 (Week 1: 53.3 ± 16.1 vs 41.9 ± 17.9 mL/min/1.73 m^2^; *P* = 0.001; Week 2: 55.3 ± 17.6 vs 46.0 ± 15.1 mL/min/1.73 m^2^; *P* = 0.008) (Figure 2A). However, this between-group difference diminished thereafter, and eGFR values gradually converged to comparable levels over time. Moreover, the LMM showed a significant group-by-time interaction for serum creatinine (*P* = 0.031). The recipients in the daprodustat group exhibited a greater decrease in serum creatinine from baseline than the control group at Day 1 (−3.97 ± 1.53 vs −2.71 ± 2.69 mg/dL; *P* < 0.001) and Day 7 (−6.32 ± 0.38 vs −5.57 ± 1.75 mg/dL; *P* = 0.013) after transplantation (Figure 2B). The incidence of SGF was significantly lower in the daprodustat group than in the control group (daprodustat 0.0% vs control 12.5%; *P* = 0.039). No cases of DGF were observed in either group.

### Clinical and graft outcomes after transplantation

No deaths, graft losses, or thrombotic events occurred during the follow-up period. Kidney biopsy findings were comparable between the daprodustat and control groups, with no significant differences in graft rejection. Protocol biopsies at 3 months post-transplantation were available for 29 patients in each group, showing comparable outcomes (*P* = 0.730). Acute rejection occurred in 1 patient (3.5%) in the daprodustat group and in none in the control group, while borderline changes were observed in 4 (13.8%) and 6 patients (20.7%), respectively; most patients showed no evidence of rejection (24 [82.8%] vs 23 [79.3%]). At 1 year post-transplantation, protocol biopsies were performed in 31 patients in the daprodustat group and 30 in the control group, and outcomes remained comparable (*P* = 0.808). Acute rejection was observed in 1 patient (3.2%) in the daprodustat group and 2 patients (6.7%) in the control group, while borderline changes were identified in 7 (22.6%) and 5 (16.7%); no evidence of rejection was observed in 23 (74.2%) and 23 (76.7%), respectively. Some patients were unable to undergo kidney biopsy due to the COVID-19 pandemic.

### Laboratory data after transplantation

The AUC of NLR from baseline (Day 0) to Day 7 was significantly lower in the daprodustat group than in the control group (*P* = 0.029) (Figure 2C and D). There were no significant differences in hemoglobin levels (Figure 2E) and red blood cells (Figure 2F) between groups after transplantation.

## Discussion

This study investigated the impact of perioperative daprodustat use on posttransplant graft function in LDKT recipients. Our findings suggest that daprodustat use might be associated with improved early graft function and a lower incidence of SGF, along with reduced early inflammatory markers. No increase in thrombotic events was observed over 1 year of follow-up. These results raise the possibility of nephroprotective benefits of perioperative daprodustat use in LDKT.

Multiple studies have reported that poor early graft function after LDKT is associated with worse long-term graft function and survival.^5^, ^6^, ^7^ Importantly, SGF, even in the absence of DGF, has been shown to increase the risk of rejection and impair graft survival.^6^^,^^7^ Taken together, the observed associations between perioperative daprodustat use, improved early graft function, and a reduced incidence of SGF raise the possibility that daprodustat may influence long-term graft outcomes, although this was not assessed in the present study. These findings warrant further investigation in larger cohorts with longer follow-up.

An animal IRI model demonstrated that HIF-PH inhibitors exert nephroprotective effects when administered before ischemia, but not after.^20^ In the present study, daprodustat was administered only to LDKT recipients, and thus the drug reached the graft at the time of reperfusion, after ischemic injury had already been initiated. Therefore, the observed improvements in early graft function associated with perioperative daprodustat use, which are suggestive of a nephroprotective effect, may not be explained solely by direct mitigation of IRI. Alternative mechanisms, such as modulation of posttransplant immune responses or attenuation of immunosuppressant-related nephrotoxicity, may also contribute.

IRI activates the immune system, triggering inflammation and increasing rejection risk.^2^^,^^3^ Roxadustat has been shown in animal studies to alleviate renal IRI via anti-inflammatory effects.^21^ Therefore, we assessed the anti-inflammatory effect of daprodustat using NLR, a marker of inflammation associated with poor renal outcomes and acute rejection.^19^^,^^22^ In this study, AUC of NLR early after transplantation was significantly lower in the daprodustat group, suggesting suppression of immune activation by IRI mitigation. However, corticosteroid effects cannot be fully excluded, as 3 recipients in the daprodustat group and 5 in the control group received off-protocol high-dose intravenous corticosteroids for 1 to 3 days within the first week posttransplant, which are known to increase NLR.^23^ This potential confounder warrants further investigation. Moreover, since NLR is not kidney-specific, future studies should include kidney-specific inflammatory markers.

TAC, a calcineurin inhibitor (CNI) used to suppress rejection, is known to induce nephrotoxicity, leading to kidney fibrosis and a decline in transplanted kidney function.^24^ Because TAC was used in all cases in this study, the possibility of its nephrotoxicity cannot be ruled out. CNIs cause renal vasoconstriction, resulting in local hypoxia and ischemia that contribute to nephrotoxicity.^24^ In this study, daprodustat may have alleviated TAC-induced renal ischemia. Supporting this hypothesis, preclinical data indicate that daprodustat can prevent CNI-induced peritubular capillary loss.^25^ Additionally, HIF-PH inhibitors have been reported to suppress kidney fibrosis through anti-inflammatory and anti-fibrotic pathways.^21^^,^^26^ These mechanisms may have mitigated TAC-induced nephrotoxicity.

This study had several limitations. First, its retrospective, single-center design may limit causal inference and generalizability. Second, the sample size was relatively small, and the study was not powered to assess definitive long-term outcomes such as graft survival or rejection. Third, despite efforts to control confounding factors through PSM, unmeasured confounders cannot be ruled out. Fourth, although no significant differences in baseline renal function were observed between the two groups after PSM, this parameter could not be fully adjusted for in the present retrospective design.

## Conclusions

Perioperative daprodustat use may be associated with improved early graft function, a reduced incidence of SGF, and lower early inflammatory markers, without an apparent increase in thrombotic risk in LDKT recipients. Further validation through large-scale, multicenter prospective studies, including randomized controlled trials, is necessary to elucidate the long-term clinical impact of daprodustat.

## Funding

This work was supported by JSPS KAKENHI (grant numbers JP26K18499 and JP25K10066).

## Author Contributions

T.S. conceptualized and designed the study, performed and analyzed the data, interpreted the results, and wrote the initial draft of the manuscript. S.T. designed the study, performed and analyzed the data, interpreted the results, and assisted in the preparation of the manuscript. H.N. and K.K. contributed to the data interpretation and revised the manuscript. T.O. contributed to data interpretation. T.H. and M.U. supervised the study and revised the manuscript. All authors have critically reviewed and approved the final manuscript.

## Data Availability

The data supporting the findings of this study are available on request from the corresponding author, subject to ethical approval. The data are not publicly available because of privacy and ethical restrictions.

## Declaration of competing interest

The authors declare that they have no known competing financial interests or personal relationships that could have appeared to influence the work reported in this article.
